# Reimagining laboratory‐based immunology education in the time of COVID‐19

**DOI:** 10.1111/imm.13369

**Published:** 2021-06-06

**Authors:** Thomas S. Wilkinson, Rob Nibbs, Nigel J. Francis

**Affiliations:** ^1^ Swansea University Medical School (SUMS) Swansea UK; ^2^ School of Life Sciences College of Medical, Veterinary and Life Sciences University of Glasgow Glasgow UK

**Keywords:** higher education, immunology education, immunology toolkit, laboratory based teaching

## Abstract

The pandemic has brought challenges to teaching lab and research skills. Here Nigel Francis and colleagues explore the diverse approaches taken to replace lab‐based immunology teaching, explain how networks of educators have driven this innovation and discuss the importance of retaining best practice into the future.

## Graphical abstract

COVID‐19 caused huge disruption to immunology teaching in higher education, but from the challenges faced, novel teaching tools and approaches have emerged. The question now remains how we encourage the use of this new ‘toolkit’ to enhance the future of immunology education?

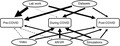

The COVID‐19 pandemic has had a profound and potentially long‐lasting effect on teaching in higher education (HE) as institutions were forced to close their physical campuses in March 2020. As SARS‐CoV‐2 swept across the globe, educators were required to move teaching online at short notice, in what became known as emergency remote teaching [1]. While some elements of education were relatively straightforward to address, such as providing live online lectures or recordings to replicate face‐to‐face (F2F) sessions, others, particularly practical classes, have been far more problematic in the new blended learning model [2]. Over a year later, uncertainty remains over when students will be allowed back into the teaching laboratory. It is therefore prudent to consider approaches that overcome the challenges associated with virtual laboratory teaching and, perhaps most importantly, ensure that the best aspects of this enforced change are retained for the future. Certainly, with the British Society for Immunology (BSI) developing a teaching resource hub (https://www.immunology.org/bsi‐teaching‐resource‐hub) there is scope to build on the educational opportunities that SARS‐CoV‐2 has presented to provide immunology educators with resources for engaging students from A‐level through to Master's‐level study. This opens up the prospect of immunology teachers developing and sharing resources to generate an immunology teaching toolkit that will have benefits to students long after the COVID‐19 pandemic has ended.

Building core sets of practical laboratory skills is a fundamental cornerstone for an immunology graduate in current times. Science‐based subjects rely on the ‘mastery’ of many complex techniques that need to be experienced ‘hands‐on’. Practical sessions or ‘laboratories’ are an opportunity for students to apply their subject knowledge and develop social skills and are a key motivator for student engagement with their course, culminating in the final‐year (capstone) research project [3]. However, under current restrictions, providing a meaningful practical learning experience or capstone opportunity is challenging. Educators are having to develop innovative ways to ‘re‐imagine’ practical class curriculums and learning objectives, ensuring that students can start to develop the core skills they will need in the workplace. Indeed, for many programmes, especially those accredited by the Royal Society of Biology (of which the British Society for Immunology is a member organization), demonstrating a range of ‘essential’ proficiencies in the laboratory is seen as a key requirement for the science graduate [3, 4]. These core skills will allow flexibility across a variety of disciplines and allow graduates to react and adapt to future challenges.

The current COVID‐19 pandemic has led to an expansion in the variety of capstone projects. These allow students to demonstrate the numerous skills they have developed during their degree, including some that are not readily demonstrable during conventional laboratory‐based projects. Dr Dave Lewis (University of Leeds) has been at the forefront of capstone project diversification, supervising a range of alternatives including ‘big data’ analyses, grant proposals, scientific communication and systematic reviews [5]. Critically, these have all been approved in the latest RSB guidelines for capstone projects [4]. Many such approaches have been used at other institutions throughout the UK, including our own. At Swansea and Glasgow University, our final‐year undergraduates, as well as postgraduate Master's and PhD students, will have benefited from perfecting non‐laboratory‐based skills in the analysis of multi‐parameter flow cytometry data, in bioinformatics and *in silico* pipeline development to understand genomic and transcriptomic data, including those generated by single‐cell RNAseq. These skills will be essential for future immunology researchers.

Nonetheless, once restrictions are lifted, it is likely that many educators will want to revert to laboratory‐based capstone projects, despite only a small proportion of students across the biosciences going on to work in a laboratory setting after graduation [6]. So, in the face of COVID‐19, how do you help students develop the skills they will need for such projects? In the early phases of a student's HE journey, the principal objectives have been to allow students to develop and demonstrate proficiencies in the laboratory, and practise data analysis, presentation and interpretation. Institutions have taken different approaches to replace laboratory classes, with some moving fully online with no face‐to‐face classes, while others have attempted to maintain laboratory‐based teaching, albeit at a vastly reduced capacity and with students working individually rather than in pairs or groups. Institutions without F2F classes are aiming to provide a theoretical understanding of the techniques, in the hope that there will be opportunity in subsequent years to demonstrate these to students in a live scenario and allow them to gain experience at a later date. In contrast, institutions providing F2F opportunities are encouraging students to gain pre‐laboratory knowledge of the rationales for using methodologies before attending laboratory classes so that they can maximize their limited hands‐on time. Across the sector, a wide range of approaches have been adopted, but they tend to focus around 4 key areas: videos, simulations, augmented reality (AR) or virtual reality (VR), and data sets. Indeed, it is clear that the diversity of teaching methods has increased during the pandemic (Figure 1) [3].

**FIGURE 1:**
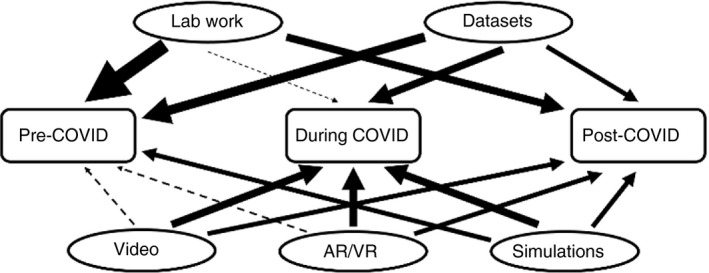
Schematic to depict the importance of different approaches adopted pre‐, during and post‐COVID‐19 to replace or support laboratory‐based teaching, which can be retained post‐pandemic to enhance the student experience and learning opportunities. Weight of line represents contribution of approach; dotted line indicates limited use. The post‐COVID‐19 provision represents a far more diverse experience for students with multiple approaches combining to enhance laboratory teaching

Videos of a variety of techniques can be found on Internet resources such as YouTube (https://www.youtube.com), LabXchange (https://www.labxchange.org), Merlot (https://www.merlot.org/merlot/) or JoVE (https://www.jove.com/education/) but with the drawback that these are not specific to the individual experiments and cannot easily be customized. Alternatively, these resources can be reproduced in‐house, if staff are able to access laboratories, and this has the added benefit that students get to see the exact equipment that they will use and the staff they are likely to see in the laboratory when circumstances permit. They can be used live to demonstrate the correct use of equipment with students watching and possibly providing input to guide the actions of the academic conducting the experiment, allowing students to make decisions that impact the experimental outcome. Alternatively, they can be pre‐recorded, with embedded or associated quiz questions to check understanding; for example, demonstrations with deliberate errors can be included for the students to identify. A slightly more complex approach, which engages students more with the learning process, is the use of interactive or branched videos. Rather than a linear experience, which can cause students to employ superficial learning approaches [7], branched videos allow students to engage with interactive elements or choose which video to watch next. One potential application of this approach is to allow students to decide on the next steps of an experiment, which includes options that result in unexpected outcomes or experimental failure. A well‐designed branched video can appear seamless to the participant but offers a tailored learning experience where the user determines their own outcome through experiential learning [8]. These resources are a wonderful virtual experience, and although there are some commercial packages available for their production (e.g. SIVA Suite), their creation often requires specialist input from a learning technologist.

The branched videos are similar to simulations, and a number of commercial companies offer immersive simulation packages, including Labster (https://www.labster.com) and Learning Science (https://learningscience.co.uk), which is used extensively at both Swansea and Glasgow. However, there is a significant cost associated with these products. Alternatively, many academics are producing simple interactive resources using coding or off‐the‐shelf software, such as Adobe Captivate. These resources can allow students to interact with websites to generate data or learn about techniques. Our COVID‐19 teaching experience at Swansea University, using Articulate Storyline 360, with the help of a dedicated learning technologist, suggests that branched videos and simulations have been very well received by second‐year undergraduate students. The development of a virtual flow cytometer simulation allowed a large second‐year laboratory class to be moved fully online, which would otherwise have not taken place due to restricted access to core research facilities during the pandemic. This coursework expanded the traditional MCQ test of previous years with three further parts: first, integration of a flow cytometry data file and software for students to ‘gate’ on leucocyte populations; second, a simulation of the selection and loading of sample tubes onto the flow cytometer, with associated ‘branched’ options where experiments can go wrong; and third, an exploration of the clinical application of flow cytometry by not only asking students to select ‘healthy’ and ‘disease’ plots from a selection of predefined immunologically distinct pathologies, such as agammaglobinaemia (no CD20^+^ B lymphocytes), but also including more challenging leucocyte profiles, such as sepsis and severe COVID‐19 to differentiate the student cohort. To date, this simulation has been delivered to over 250 students (during the pandemic) and will be retained as part of immunology provision post‐pandemic. This strategy will be expanded to other key immunology techniques including ELISA and PCR, with all resources being made freely available.

A further step‐up from simulations is the use of either virtual or augmented reality. Virtual reality (VR) is a more totally immersive experience, while augmented reality (AR) uses the digital overlay of information on a live view. Alternatively, AR allows for multiple students to view the same object from different angles simultaneously [9]. There are apps available, including ZapWorks (https://zap.works), that allow staff and students to create resources where they can interact with, for example protein structures, which can be downloaded from Protein Databanks or repositories such as SketchFab (https://sketchfab.com). Prior to the pandemic, Glasgow University was developing a suite of VR apps to supplement learning across multiple disciplines (https://www.edify.ac), including one that allowed students to travel the body during the development of an immune response to *Salmonella* infection. Fortunately, a laboratory simulation had also been developed, which, once the pandemic arrived, enabled educators to provide a simulation for more than 500 third‐year Life Science students of a quantitative PCR laboratory assessing the level of Zika virus in patient samples. Although the educator rather than the student was in the driving seat, it provided an interesting, alternative way of demonstrating laboratory techniques to a large online audience.

Perhaps the easiest and most traditional approach is the provision of data sets, either real or generated, which allows students to develop data analysis and presentation skills. These can be used to supplement hands‐on laboratory time, to allow students to focus solely on technique acquisition during F2F classes or in combination with other approaches described above to provide a fully virtual laboratory experience. Alternatively, they can be integrated into an investigative‐style project that aims to develop skills in hypothesis setting and experimental design, as well as data analysis, presentation and interpretation. At Glasgow, students starting their first of two years of immunology study (after completing 2 years of broad Life Science education) undertook a 10‐week Virtual Investigative Project (VIP) that aimed to simulate an immunology research group. Four to five students (‘the researchers’) were assigned to a principal investigator (PI) and research assistant (RA) (an educator and a graduate teaching assistant (GTA), respectively) and, across five 1‐hr meetings, met to discuss the project background, set hypotheses, design virtual experiments and discuss data. Learning Science and JoVE resources, along with technique lectures, provided students with insights into experimental techniques, while GTAs offered first‐hand knowledge of experimental design, controls, sample size, etc., and generated virtual data from the experiments the students designed. A simple low‐stakes assessment at the start of the semester, with feedback, provided early guidance on data analysis and presentation, and statistics. Students produced a virtual laboratory report at the end of the VIP in the style of a research paper and undertook some peer assessment to recognize each other's contribution to group work.

Of course, the principal disadvantage of all these approaches is that they still cannot truly replicate the hands‐on laboratory experience, where students learn the fine psychomotor skills needed to conduct laboratory work, experience the pleasure of successfully generating their own real data and develop resilience on how to cope with the frustration of failed experiments. Wherever possible, these non‐laboratory‐based approaches should be used to supplement, rather than replace, laboratory experience. However, even on their own, they can be used to allow students to understand the process of scientific investigation and develop theoretical insights and analytical skills that they can then apply and practise in the laboratory in subsequent years of their degree.

While COVID‐19 has been immensely disruptive for laboratory‐based degrees, there are many reasons to be positive. The pandemic has forced educators to think long and hard about what is important for their students to learn and how learning objectives can be met at both module and programme level. Students have been able to learn different skills that they would not have had the opportunity to learn in the past. Moreover, there has been a remarkable willingness among academics to communicate with each other and share resources and ideas. At the start of the pandemic, a number of networks were set up to share ideas for projects and general teaching, with #DryLabsRealScience focussing on Life Sciences [10, 11]. The lectuREmotely website includes a section on laboratory teaching (https://www.lecturemotely.com/labcourses) that provides videos of the webinars and other free resources, including Dave Lewis’ guide to capstone projects and short ‘how‐to’ guides for ‘getting started’ with alternative projects and free resources for a variety of topics, including immunology. There remains, however, a lack of high‐quality immunology resources that can be used to support remote teaching.

That brings us to the key question: where next? COVID‐19 could be a watershed moment for HE. The traditional model of teaching has largely remained unchanged for the past 500–600 years, and it is important that the best of teaching innovation is retained [12, 13]. Through necessity, alternatives to laboratory classes and laboratory projects have been developed, and we should retain or adapt these resources when laboratories reopen, not because we have to, but because we ought to. Non‐laboratory‐based projects have provided students with the opportunity to develop research skills that will be critical for future immunology researchers, particularly in bioinformatics and the analysis of complex data sets. They have also allowed students that might not want to go into laboratory research to showcase their talents in a variety of different ways. The pandemic has enabled students to have experienced innovative forms of learning, and developed new skills, earlier in their academic journey in a way that they would not have had in pre‐COVID times. It would be hugely disappointing to have overcome the challenges posed by the pandemic simply to revert back to what is comfortable and easy. Indeed, this would ultimately do our students a disservice because COVID‐19 has forced us to re‐evaluate our teaching, find new and innovative ways to deliver material, and focus on those things that are important for our students to learn. The HE community has shown that there is a willingness to share and finding new ways to spread the innovative ideas of immunology educators will only help strengthen the sector and provide the UK with even better immunology teaching.

## Acknowledgements

The authors acknowledge the contributions of members of the #DryLabsRealScience network for sharing their innovative ideas.

## Author contributions

All authors contributed equally to the writing, critical review and approval of the manuscript.
